# Viral Reactivation in Immunocompetent Critically Ill Patients

**DOI:** 10.3390/microorganisms14092090

**Published:** 2026-09-18

**Authors:** Konstantinos Mantzarlis, Asimina Valsamaki, Demosthenes Makris, Antonios Katsounas, Chiara Fanelli, Slavica Kvolik, Hakan Erdem, Despoina Koulenti, on behalf of the ESGCIP

**Affiliations:** 1 Faculty of Medicine, School of Health Sciences, University of Thessaly, 41500 Larissa, Greece; mantzk@outlook.com (K.M.); dimomakris@uth.gr (D.M.); 2 Department of Critical Care, University Hospital of Larissa, 41110 Larissa, Greece; semi_val@hotmail.com; 3 Division for Infectious Diseases and Critical Care, Department of Medicine, Knappschaft Kliniken University Hospital, Ruhr University, Bochum, 44892 Bochum, Germany; antonios.katsounas@knappshaft-kliniken.de; 4 Department of Medicine, Surgery, and Pharmacy, University of Sassari, 07100 Sassari, Italy; chiarastellafanelli@gmail.com; 5 Perioperative, Acute, Critical Care and Emergency Medicine Section, Department of Medicine, University of Cambridge, Cambridge CB2 0AW, UK; 6 Medical Faculty, Josip Juraj Strossmayer University, 31000 Osijek, Croatia; slavica.kvolik@gmail.com; 7 Osijek University Hospital, 31000 Osijek, Croatia; 8 Department of Infectious Diseases and Clinical Microbiology, Gülhane Training and Research Hospital, University of Health Sciences, Ankara 06010, Türkiye; erdemhakan@gmail.com; 9 King’s College Hospital NHS Foundation Trust, London SE5 9RS, UK; 10 Frazer Institute, The University of Queensland, Brisbane QLD 4102, Australia; 11 School of Health Sciences, University of Ioannina, 45500 Ioannina, Greece

**Keywords:** Viruses, Antivirals, Reactivation, Critically ill

## Abstract

The course of critically ill patients admitted to the intensive care unit very often may be complicated by viral infections. Latent viruses may reactivate in ‘apparently’ immunocompetent critically ill patients as consequence of inflammatory and immune dysregulation secondary to critical illness. Evidence suggests that reactivation correlates with worse clinical outcomes, including increased mortality. Highly sensitive molecular diagnostic techniques have enhanced the ability to detect viral pathogens, but still consensus definitions for reactivation and subsequently the discrimination between reactivation and overt infection in critically ill patients are lacking. Despite the progress in detecting viral pathogens, their role in clinical outcomes is obscured. Furthermore, the role of antiviral therapy, either prophylactically/preemptively or as treatment after virus detection, has to be further investigated. The question of whether reactivation is the cause or a surrogate marker of underlying severity of illness has not yet been fully elucidated.

## 1. Introduction

Critically ill patients admitted to the intensive care unit (ICU) very often require mechanical ventilation and/or vasoactive agents, and multiorgan support [1]. Viral infections may complicate the clinical course of critically ill patients, resulting in worse outcomes [2]. Besides de novo infections, certain latent viruses may reactivate in both immunosuppressed and immunocompetent critically ill patients, i.e., without overt immunosuppression according to the classic EORTC/MSG host factors of immunosuppression (Table 1) [3,4]. Non-classical factors that lead to a varying degree of immunosuppression in critical illness are presented in Table 2 [5,6,7,8,9,10]. The advent of highly sensitive molecular diagnostic techniques has enhanced our ability to detect viral pathogens. The incidence of such infections has unambiguously increased in recent years [11]; this trend may reflect improved diagnostics, but also a true rise in the number of vulnerable patients admitted to the ICU [12]. Despite progress in detecting viral pathogens, our understanding of their role in clinical outcomes—and when antiviral treatment should or should not be initiated—remains limited; this knowledge gap might contribute to poor outcomes.

Innate and adaptive immune responses play a pivotal role in controlling viral infections. During the primary viral infection, the recognition of viral pathogen-associated molecular patterns (PAMPs) by the host cells’ pathogen recognition receptors (PRRs) triggers signaling pathways that induce pro-inflammatory cytokines, particularly interferons (IFNs), and promotes the migration of innate immune cells to the site of infection [13]. Antigen presentation activates CD4+ helper T cells, which in turn induce both CD8+ T cell and B cell responses, resulting in specific antibody production. After the primary infection, viruses belonging to the *Herpesviridae* family become dormant in neurons, i.e., varicella zoster virus [VZV (HHV-3)], and herpes simplex virus 1 (HSV-1) and 2 (HSV-2), in B cells, i.e., Epstein–Barr virus [EBV (HHV-4)] and myeloid cells, i.e., cytomegalovirus [CMV (HHV-5))]. Viral reactivation may occur in case of severe illness due to inflammatory and immune dysregulation. Inflammatory and immune dysregulation secondary to severe illness may lead to viral reactivation. For example, CMV reactivation can be triggered by several factors [14]. Cytokines such as tumor necrosis factor-a (TNF-a) and interleukin-1β (IL-1β) may drive viral replication, as TNF-a can directly activate CMV gene expression. Additionally, endotoxins and catecholamines may induce similar effects [15,16,17]. Immune deficiency resulting from critical illness further compounds this risk. The role of T cell immunity [18], natural killer (NK) [19], and dendritic cells’ function [20] is crucial.

In this narrative review, we present and critically discuss up-to-date data on epidemiology, pathogenesis, clinical impact and treatment of the reactivation of the most common members of the herpesvirus family in immunocompetent critically ill patients, i.e., ICU patients without overt immunosuppression.

## 2. Cytomegalovirus (CMV (HHV-5))

**Virology:** CMV is a member of the human herpesvirus family, and one of the largest double-stranded DNA viruses known to cause clinical disease. CMV is a widespread virus; humans and other primates serve as natural hosts, and once infected, the body retains the virus for life, since CMV remains quiescent in monocytes and macrophages in multiple organs, a state defined as latency [21].

**Epidemiology:** A survey in the United States reported an overall CMV seroprevalence rate of 50.4% [21]. CMV infection is among the most prevalent causes of opportunistic infection in patients with impaired immunity and is particularly common in recipients of allogeneic hematopoietic stem cell transplants or solid organ transplants [22].

**Pathogenesis:** Reactivation of the dormant virus is the most common mechanism of infection in such patients. It is more frequent than CMV transmission through blood product transfusions. There are no specific definitions for virus reactivation or infection in immunocompetent patients. The only available definitions refer to transplanted patients. According to the consensus definition, CMV infection is defined as the isolation of the virus by culture, or detection of viral proteins (antigenemia) or nucleic acid (DNAemia), regardless of symptomatology. However, to qualify for CMV disease, clinical symptoms attributable to CMV infection should be present. The existence of proven end-organ disease is a subdivision of CMV disease called tissue-invasive disease [23].

The injurious mechanisms of CMV reactivation are not well elucidated [24]. Three major mechanisms have been proposed. The first one suggests that the virus has a direct cytopathologic effect that causes damage to specific organs [25]. The second mechanism proposes that virus reactivation causes an immunopathological effect, in which damage to tissues is caused by the excessive immune response as a defense to the virus (inflammatory damage) [26]. Finally, the third mechanism is related to an alteration in the immune system, rendering patients more susceptible to secondary fungal and bacterial infections [27,28,29].

**Definitions of Reactivation:** There are several unanswered questions regarding CMV reactivation/infection in critically ill immunocompetent patients. The first issue is that there is no consensus definition for CMV reactivation and the discrimination between reactivation and overt infection in critically ill, ‘apparently’ immunocompetent patients. It is unknown if there is a threshold of viremia that is harmful. Several definitions and viremia thresholds have been used in different studies; the choice of viral cultures, antigenemia or positive polymerase chain reaction (PCR) for the virus detection may impact the reported incidence of CMV reactivation/infection. Consequently, definite conclusions remain elusive.

**Impact on Clinical Outcomes:** Several studies provide data regarding the reactivation of the CMV in critically ill immunocompetent patients and its clinical significance. In general, the results are indicative of a relationship between CMV reactivation and worse outcomes.

In one of the first studies in the field, 40 ICU patients with CMV antigenemia without overt immunosuppression were compared with 40 ICU patients without CMV antigenemia, matched for age, gender, disease severity and cause of admission. CMV reactivation was defined as a cumulative incidence of viremia greater than 1000 copies/mL. Patients with CMV presented longer duration of mechanical ventilation (35 ± 27 vs. 24 ± 20 days, *p* = 0.02), longer ICU stay (41 ± 28 vs. 31 ± 22 days, *p* = 0.04), and higher mortality (50% vs. 28%, *p* = 0.02) [30]. CMV was detected more often after a delay of 10 days after hospital admission and was significantly correlated with renal failure and corticosteroid therapy during ICU stay [30]. In another prospective study the results were similar [31]. CMV plasma DNAemia was assessed by thrice-weekly real-time PCR and clinical outcomes of 120 CMV-seropositive ‘apparently’ immunocompetent adults were analyzed. CMV seropositivity, indicating previous CMV infection, was defined by the detection of anti-CMV antibodies using a commercial enzyme immunoassay kit. Logistic regression analysis indicated that CMV infection was an independent predictor of hospitalization or death within 30 days. At any level of DNAemia, the adjusted odds ratio (OR) was 4.3 (95% CI: 1.6–11.9, *p* = 0.005). When DNAemia exceeded 1000 copies/mL, the OR increased to 13.9 (95% CI: 3.2–60, *p* < 0.001). In addition, a recent multicenter study of 410 sepsis patients reported that latent CMV infection (positive serology) was linked to significantly higher 30-day mortality (38% vs. 25% in seronegative patients; HR~1.65). CMV-seropositive patients also exhibited distinct immune profiles—for instance, elevated interleukin 6 (IL-6) predicted outcomes only in this group—suggesting that pre-existing CMV latency may worsen immune dysregulation during critical illness [32].

Chiche et al. [27] found that several factors were significantly associated with an increased risk of active CMV infection in ICU patients: prior admission to other hospital wards (odds ratio [OR] 2.49, *p* = 0.043), blood transfusions (OR 3.31, *p* = 0.04), enteral feeding (OR 3.00, *p* = 0.005), recent corticosteroid use before ICU admission (OR 2.26, *p* = 0.08), and older age (OR 1.026, *p* = 0.07). Active CMV infection was defined by one or more of the following: positive CMV pp65 antigenemia (≥1 cell), positive bronchoalveolar lavage (BAL) shell vial culture, histological evidence of CMV infection, or the presence of pulmonary symptoms with CMV detected in BAL fluid or lung tissue. Patients with active CMV infection had higher ICU mortality (54% vs. 37%, *p* = 0.082) and in-hospital mortality (59% vs. 41%, *p* = 0.058) compared to those without infection. Multivariate analysis confirmed an association between CMV infection and ICU deaths. Additionally, these patients experienced longer duration of mechanical ventilation and extended ICU stays, and were significantly more likely to develop bacterial nosocomial infections (69% vs. 33%, *p* < 0.001) [27].

Another factor that may contribute to CMV reactivation is sepsis. In a study examining viral reactivation in patients with sepsis [29], plasma samples were obtained from 560 critically ill septic patients, 161 critically ill non-septic patients, and 164 healthy age-matched patients, and analyzed by real-time PCR for CMV and other viruses. The study aimed, firstly, to test the hypothesis that the reactivation of latent viruses occurs with prolonged sepsis, thereby providing evidence of relevant sepsis-induced immunosuppression, and secondly, to test if markedly elevated viral loads contribute to morbidity and mortality [29]. The results showed that septic patients with viremia compared to septic patients without viremia had increased fungal and opportunistic bacterial infections. Patients with detectable CMV DNA in plasma had higher 90-day mortality compared to CMV-negative patients. Overall, there was a 24.2% incidence of CMV reactivation. The authors concluded that reactivation of latent viruses, including CMV, is very common in patients with sepsis, and this finding is consistent with the development of immunosuppression. However, it cannot be confidently determined whether reactivated viruses contribute to morbidity and mortality or are simply a secondary manifestation of the course of the disease. Similar findings were reported in another study by Heininger et al. [33] that reported a high percentage of patients (40.7%) with severe sepsis presenting with CMV reactivation, but mortality rates in patients with and without CMV reactivation were similar (37.1% vs. 35.3%, *p* = 0.861). However, CMV reactivation was independently associated with increased length of stay in the ICU (HR 3.365; 95% CI 1.233–9.183, *p* = 0.018) and in the hospital (HR 3.3, 95% CI 1.78–6.25, *p* < 0.001), as well as prolonged mechanical ventilation (HR 2.6, CI 95% 1.39–4.94; *p* < 0.001) and impaired pulmonary gas exchange (*p* = 0.038). The relationship between the severity of the disease and CMV reactivation was confirmed by another study in which 80 patients with CMV reactivation (defined as CMV plasma DNAemia ≥ 500 copies/mL) had higher sequential organ failure assessment (SOFA) scores compared to patients without reactivation during the entire 28-day observation period [34]. The median time of CMV reactivation was day 7 post-ICU admission [34]. Also, the total number of red blood cell units transfused and C-reactive protein (CRP) levels upon ICU admission were independently associated with CMV reactivation. Nevertheless, mortality, length of ICU stay, and duration of mechanical ventilation were similar in the two groups [34]. On the other hand, in a large three-ICU cohort study of 431 COVID-19 acute respiratory distress syndrome (ARDS) patients, CMV reactivation occurred in ~20% and was linked to more severe illness and higher unadjusted mortality (67% vs. 24% in non-reactivators). However, after adjusting for illness severity and risk factors, CMV viremia was not an independent predictor of mortality, underscoring the ongoing debate over whether CMV is merely a marker of critical illness or a causal contributor to worse outcomes [35]. Finally, in a study by Ong et al., CMV reactivation was examined in the specific population of ‘apparent’ immunocompetent patients who presented with ARDS [36]. Of 399 ARDS patients, 271 (68%) were CMV-seropositive and reactivation occurred in 74/271 (27%); CMV reactivation was associated with increased ICU mortality (adjusted sub distribution hazard ratio (SHR) 2.74, 95%CI 1.51–4.97) and population-attributable fraction of ICU mortality by day 30 of 23% (95% CI 6–41). This result differs slightly from a previous study from the same lead author [37], which also associated CMV reactivation in patient with ARDS with worse outcomes, particularly prolonged mechanical ventilation and increased mortality, but only in the subgroup of patients with septic shock, while patients suffering from ARDS without septic shock and CMV seropositivity had similar outcomes to the control group.

Three randomized trials tested the safety and efficacy of antiviral treatment for the prevention or treatment of CMV infection in critically ill immunocompetent patients admitted to the ICU. The first trial [38] was a single-center, open-label, randomized, controlled clinical trial, where 124 CMV-seropositive patients indicating previous exposure to CMV were randomized in three groups: the first group received anti-CMV prophylaxis with valacyclovir hydrochloride (n = 34), the second received low-dose valganciclovir hydrochloride (n = 46), and the third had no intervention (n = 44). The primary outcome was time to first CMV reactivation in blood, which was defined as detected CMV DNA copies above the lower limit of the quantitative PCR assay (20 copies/mL) from initiation of the study drug until day 28. Although recruitment into the valacyclovir arm stopped prematurely due to increased mortality in comparison to the other two groups, combined treatment groups presented a lower rate of CMV reactivation [hazard ratio (HR), 0.14; 95% CI, 0.04–0.50; *p* = 0.002 for combined treatment group vs. control]. Other outcomes, i.e., clinical, safety, and exploratory cytokine outcomes, were similar between the groups. Similar results were reported in another study [39], a double-blind, placebo-controlled, randomized clinical trial with a longer follow-up of 180 days. This study included 160 non-immunocompromised adults hospitalized for severe sepsis or trauma and respiratory failure. CMV-seropositive adults were randomized to receive either prophylactic ganciclovir or placebo [39]. The primary outcome was to determine whether ganciclovir prophylaxis reduces plasma IL-6 levels from day 1 to 14. Secondary outcomes included: incidence of CMV reactivation in plasma, mechanical ventilation days, incidence of secondary bacteremia or fungemia, ICU length of stay, mortality, and ventilator-free days (VFDs) at day-28. The study found no statistically significant differences in IL-6 levels change between the groups. Among the secondary outcomes, prophylactic ganciclovir was associated with significantly lower rates of both low-grade and high-grade CMV reactivation (>1000 IU/mL) in plasma and with a greater number of ventilator-free days (VFDs). Finally, in the most recent study on CMV treatment [40], ganciclovir was administered to mechanically ventilated patients in a preemptive manner, and not as prophylaxis, unlike in previous studies, as the patients presented with CMV reactivation defined as “DNAemia” with a threshold of 500 IU/mL in whole blood. The primary outcome was VFDs from randomization to day 60 compared with the control group. The trial was stopped for futility, as the interim analysis showed no significant differences between the groups [patients receiving ganciclovir (N = 39) compared with control patients (N = 37)], neither for being alive and weaned from mechanical ventilation at day 60 (HR, 1.14; 95% CI 0.63–2.06; *p* = 0.66) nor for VFDs (median [IQR] numbers of VFDs for ganciclovir-treated patients and controls were 10 [0–51] and 0 [0–43] days, respectively, *p* = 0.46). Mortality at day 60 was 41% in patients in the ganciclovir group and 43% in the placebo group (*p* = 0.845). The authors concluded that in patients mechanically ventilated for ≥96 h with CMV reactivation in blood, preemptive ganciclovir did not improve the outcomes.

In their most recently published paper, Cannac et al. (2025) [41] stated that CMV reactivation in mechanically ventilated patient occurred in 34.7% of SARS-CoV-2-negative patients and in 45.4% of SARS-CoV-2-positive patients and that treatment with methylprednisolone was statistically significant associated with CMV reactivation (*p* < 0.001). Among patients with reactivation, administration of ganciclovir was a protective factor for 60-day mortality (*p* = 0.004). In the same year, Kim et al. reported a 45% incidence of CMV reactivation in the lower respiratory tract of critically ill patients, which was associated with increased ICU, in-hospital, 30-day, and 90-day mortality. However, anti-CMV therapy was not associated with overall survival [42]. Finally, Çimendağ et al., in a prospective observational study of 146 immunocompetent patients in the ICU, reported an incidence of 17.8% reactivation, with a mean onset of 10 ± 4.72 after admission. Factors independently associated with CMV reactivation were sepsis, the APACHE II score value at ICU admission and the duration of the illness before admission. The incidence of fungemia after ICU admission was statistically significantly higher in the CMV reactivation group [43].

**Immune Mechanisms and Potential Pathways of Viral Reactivation:** Persistent inflammation, immunosuppression, and catabolism syndrome (PICS) is characterized by the coexistence of persistent inflammation and acquired immune dysfunction [10,44]. The expansion of myeloid-derived suppressor cells (MDSCs) is considered an important mechanism contributing to PICS-associated immunosuppression, including impaired adaptive T-cell responses [10,44]. This immunosuppressive state may facilitate reactivation of latent herpesviruses during critical illness [29].

**Conclusions:** It is still not clear whether CMV positivity is a surrogate marker of underlying disease severity or represents a real pathogen that causes infection leading to worse outcomes. The fact that studies adjusting for baseline illness severity presented a correlation between CMV reactivation and worse outcomes suggests that CMV reactivation might be a real threat to critically ill patients. On the other hand, administration of antivirals, either prophylactic for seropositive patients to prevent CMV reactivation, or as preemptive therapy initiated at any level of viremia, has not demonstrated consistently favorable clinical outcomes.

## 3. Herpes Simplex Virus 1, 2 (HSV 1,2)

**Virology:** HSV is a DNA virus from the *Herpesviridae* family, commonly classified into two types—HSV-1, typically associated with oropharyngeal infections, and HSV-2, linked to genital infections. However, both types can lead to severe systemic manifestations in immunocompromised or critically ill immunocompetent patients [45] after potential reactivation under conditions of stress and mechanical ventilation. This type of reactivation might cause secondary bacterial infections, encephalitis, or HSV pneumonia, which can complicate patients’ clinical course. Early detection, prompt therapy, and proper management are key to reducing the impact of herpetic infections on morbidity and mortality [45,46,47].

**Epidemiology:** HSV infections of the lower respiratory tract in adult population are not common and have been reported almost exclusively in patients with severe immunosuppression. Hraiech et al. reported a range of 5–64% for HSV reactivation, depending on the diagnostic method used [45].

**Definitions of Reactivation:** The identification of HSV reactivation relies heavily on the use of sensitive and specific tools, which remain a critical focus in both clinical and research settings. However, there is still no consensus on the definition for HSV reactivation. It is again unknown if there is a threshold of viremia that is harmful. A study by De Vos [48] highlighted the use of real-time PCR for quantifying HSV DNA in lower respiratory tract samples, offering a potentially reliable method to distinguish between active infection and viral shedding. The study showed that HSV reactivation typically begins around seven days post-intubation and follows a pattern of exponential viral growth, underscoring the importance of timely diagnostics and monitoring.

**Impact on Clinical Outcomes:** Several studies outlined a significant prevalence of HSV reactivation and its association with adverse clinical outcomes. Costa et al. [49] stated that a viral load of ≥10^5^ copies/mL in the bronchoalveolar lavage fluid was associated with worsening clinical features, such as compromised immune status, the need for mechanical ventilation and admission to ICU, occurrence of co-infection, and more frequent occurrence of death with 21 days.

Sundar [50] described that HSV-1 reactivation in 48 ICU patients (mixed population: elderly septic with ARDS, post-thoracotomy and immunosuppressed) in their respiratory secretions led to complications such as hemorrhagic respiratory secretions, mucosal ulcers, and secondary infections, which contributed to the extended duration οf mechanical ventilation and increased mortality rate. More specifically, the authors stated that HSV-1 reactivation was associated with 52% occurrence of VAP, and a significant rate of 42% mortality. Likewise, it was observed that HSV found in bronchoalveolar lavage fluid (BALF) is highly linked to ventilator-associated pneumonia (VAP) and other complications from the respiratory system. These results support previous findings that connected immunological dysregulation, systemic inflammation, and multi-organ failure to HSV reactivation [51].

Existing and emerging data suggest reactivated HSV can act as a pathogen or co-pathogen in the setting of critical illness. For example, a single-center study reported HSV-1 reactivation in nearly half of invasively ventilated COVID-19 patients; those experiencing HSV reactivation had higher 60-day mortality (58% vs. 33%) and higher rates of VAP. HSV-1 reactivation was identified as an independent risk factor for death in this cohort (adjusted HR~2.0), highlighting how virus reactivation, enabled by profound inflammation and steroid-induced immunosuppression, may worsen outcomes in otherwise immunocompetent ICU patients [52]. On the other hand, Luyt et al. [53], in an older prospective single-center observational study, aimed to define the frequency, risk factors and relevance of HSV bronchopneumonitis in ICU patients with prolonged invasive mechanical ventilation. All consecutive immunocompetent patients mechanically ventilated for more than five days were evaluated; HSV bronchopneumonitis was diagnosed in 21% (42/201) of the patients that clinically deteriorated after a mean duration of mechanical ventilation of 14 days (+/−6), with identified risk factors being the presence of oral-labial lesions, identification of HSV in the throat, and the presence of macroscopic lesion during bronchoscopy [53]. The patients that developed HSV bronchopneumonitis had higher morbidity, with more complications and longer duration of mechanical ventilation and ICU stay [53]. However, further evidence is needed to establish a robust causal link between mechanical ventilation and HSV reactivation and its role as a pathogen/co-pathogen.

In a double-blind RCT across 16 ICUs (238 patients) preemptive acyclovir administration was tested in mechanically ventilated, immunocompetent patients with HSV-1 oropharyngeal reactivation. The trial found no significant improvement in outcomes—acyclovir did not increase 60-day ventilator-free days and did not significantly reduce 60-day mortality (22% vs. 33% in placebo, *p* = 0.06). These results do not support routine prophylactic acyclovir for HSV in ICU patients, indicating that HSV reactivation may often be an epiphenomenon rather than a treatable driver of poor outcomes [54]. The clinical impact of HSV reactivation in ICU patients was studied by Linssen [51] and Costa [49]. Reactivation rates were 32% and 31%, respectively, in distal airways among patients suspected of developing VAP. More specifically, in Linssen et al.’s study [51], HSV-1 DNA was detected in 4.3% of patients admitted to the hospital <48 h before bronchoalveolar lavage, 15% of non-ICU patients and 32% of ICU patients. The authors observed the highest mortality in patients with HSV-1 load > 10^5^ copies/ml. According to this correlation, HSV reactivation may raise the risk of developing secondary bacterial or fungal infections, which in turn may lead to pulmonary complications. HSV has been incriminated for worsening ARDS or contributing to sepsis, a fact that further emphasizes its clinical relevance in ICU settings. These studies collectively indicate that HSV reactivation may not merely be a marker of critical illness but an active driver to poor outcomes, including prolonged ICU stay, higher mortality rate, and increased risk of organ failure.

The possible beneficial effects of antiviral therapy for ICU patients with HSV reactivation have been the subject of recent studies. A comprehensive review and meta-analysis [55] examined the effects of antiviral therapy, including acyclovir, on patients on mechanical ventilation whose respiratory samples were positive for HSV. The study reported that antiviral therapy was associated with reduced hospital mortality (52.7% to 40.6%, RR 0.74 [0.64–0.85]) and reduced 30-day mortality (RR 0.75 [0.59–0.94]). No significant effect on ICU mortality was observed (RR 0.73 [0.51–1.05]). However, the overall quality of evidence was low due to study design limitations and small sample sizes. These results indicate a potential therapeutic benefit of antivirals, but confirmation through larger, rigorously designed trials is required.

**Conclusions:** HSV reactivation in immunocompetent ICU patients is a potentially significant contributor to complications such as prolonged ventilation, secondary infections, and increased mortality. While antiviral treatments like acyclovir have shown promise, their routine use is still subject to further validation through high-quality trials. Combined with advanced diagnostic tools such as real-time PCR, these interventions have the potential to improve outcomes for critically ill patients by enabling earlier detection and more effective management of HSV reactivation. The evidence from recent reviews emphasizes the need for a proactive approach to mitigate the clinical burden of HSV in ICU settings.

## 4. Epstein–Barr Virus (EBV (HHV-4))

**Virology:** EBV, a member of the *Herpesviridae* family, is an enveloped, icosahedral, double-stranded DNA virus (172 kb genome), known for its ability to establish lifelong latency after primary infection [56]. It belongs to the human herpesvirus type and is the first identified oncogenic virus, establishing permanent infection in human B cells. For critically ill patients, without overt immunosuppression, EBV reactivation is a significant concern, particularly in those with prolonged ICU stay, and is often linked to systemic inflammation, immune dysregulation, and poor outcomes, including secondary infections and increased mortality [56].

**Epidemiology:** It is estimated that >90% of the adult population have been infected with the virus at some point of their lives, and the virus is implicated in about 1.5% of all cancers worldwide [57].

**Definitions of Reactivation:** Recent studies have placed an emphasis on the diagnostic value of EBV DNA measurement [56,58,59,60]. Detecting patients at risk of serious consequences requires the ability to distinguish between latent viral presence and active viral replication, which is made possible by real-time PCR monitoring of viral loads. EBV viral load is a significant tool for prognostic and therapeutic decision-making since it corresponds with the severity of illness and the probability of unfavorable outcomes, as highlighted by several studies [58].

**Impact on Clinical Outcome:** EBV reactivation, which seems to be linked to the host’s immune dysregulation, is often exacerbated by prolonged mechanical ventilation, corticosteroid use, or other immunosuppressive therapies [59,60]. EBV DNA was found in the blood of 71% of EBV-seropositive patients who stayed in the ICU for more than five days [58]. Mortality was higher among patients with EBV reactivation (33/61 vs. 7/25, *p* = 0.02); moreover, patients’ total length of stay and duration of mechanical ventilation were longer (21 vs. 10 days, *p* < 0.001 and 15 vs. 7 days, *p* < 0.001 respectively). Moreover, studies have shown that EBV reactivation can lead to secondary bacterial or fungal infections, further increasing morbidity and mortality rates. In the study by Libert [58], it was found that EBV reactivation correlated with higher rate of secondary infections, except for the longer ICU stay and the increased rate of organ failure. An explanation may be that EBV reactivation is associated with significant inflammatory responses, such as the cytokine storm frequently seen in ARDS or septic shock. EBV reactivation exacerbates systemic inflammation by disrupting the host’s immune equilibrium, which can aggravate pre-existing conditions like ARDS. Studies further corroborate this link, suggesting that *Herpesviridae* reactivation, including EBV, plays a role in pulmonary complications, potentially worsening respiratory failure or ARDS outcomes.

Therapeutic options for managing EBV reactivation remain limited. While antiviral therapies such as ganciclovir have been tested in several studies as prophylaxis or treatment for other herpesviruses like CMV, their efficacy in treating EBV reactivation in ICU settings is less well-documented since data are unavailable. During latent EBV infection, lytic-phase viral kinases required for efficient activation of nucleoside analogs are not expressed, which markedly limits the activity of ganciclovir against latent infection [56]. Newer antivirals such as maribavir, currently licensed for the treatment of refractory or resistant CMV infection, have also shown efficacy against viral EBV DNA replication, interfering with the activity of the viral protein kinase BGLF4; however, further in vivo studies are needed to assess if they have any efficacy against EBV reactivation and any future role in the ICU setting [61,62]. Interestingly, another antiviral, letermovir, used for CMC prophylaxis, has been associated with an increased incidence of EBV reactivation although the underlying mechanisms remain unclear [63,64].

## 5. Varicella Zoster Virus (VZV (HHV-3))

**Virology:** The herpesvirus family VZV is responsible for two different illnesses: chickenpox as initial infection, and herpes zoster when the virus is reactivated. In the dorsal root ganglia, the virus develops latency following the initial infection and reactivates in response to stress or immunosuppression. VZV is an enveloped, double-stranded DNA virus with icosahedral symmetry and a linear DNA genome of about 125 kb, encoding 71 proteins [65].

**Epidemiology:** In 2019, an estimated 83,963,744 incident VZV infections and 14,553 associated deaths occurred globally. From 1990 to 2019, the age-standardized incidence rate increased slightly, whereas age-standardized mortality and disability-adjusted life year (DALY) rates decreased [66].

**Definitions of Reactivation:** No consensus definition for VZV reactivation in critically ill patients is available.

**Impact on Clinical Outcomes:** VZV reactivation presents manifestations such as pneumonia, encephalitis, and systemic dissemination, which often complicate the clinical course. In addition to immunocompromised patients that are at higher risk for reactivation, immunocompetent ICU patients have been shown to exhibit VZV reactivation [67]. This makes VZV a significant concern, requiring timely diagnosis and intervention. Reactivation of VZV in ICU patients is associated with substantial morbidity, including systemic infections and respiratory complications. Data are scarce. Hagiya et al. [67] reported systemic VZV infections in two ICU patients, one with severe acute pancreatitis and another with purpura fulminans. Both patients experienced respiratory failure and other complications, including disseminated VZV lesions; even after taking acyclovir intravenously, one patient died.

In a multicenter study [68] by Mirouse et al., data from 102 patients with VZV-related community-acquired pneumonia (VZV-CAP) that were admitted to the ICU were analyzed. This cohort concerned severe VZV-CAP rather than endogenous VZV reactivation. The patients in this cohort were young (mean age 39 years) and 52% of them were immunocompromised. The mean time since respiratory symptom onset was 2 days (1–3 days). Most patients required mechanical ventilation, with ARDS diagnosed in 80% of patients. The study found that early bacterial co-infections and a higher SOFA score significantly increased the need for intubation and mechanical ventilation. Although all patients received acyclovir, hospital mortality was 24%, indicating the severity of the condition.

Regarding antiviral treatment, the American Society of Health-System Pharmacists (ASHP), recommends IV acyclovir (10–15 mg/kg every 8 h) as standard therapy for hospitalized patients with serious VZV infections [69]; nevertheless, the efficacy of adjunctive treatments, such as corticosteroids, remains inconclusive. In the study of Mirouse [68], detailed previously, corticosteroid use was associated with increased the risk of secondary infections without reducing mortality, raising questions about their role in management. Early identification of high-risk patients and prophylactic measures, including vaccination for healthcare workers and susceptible patients, may mitigate the burden of VZV in ICUs.

## 6. Conclusions

**Figure 1:**
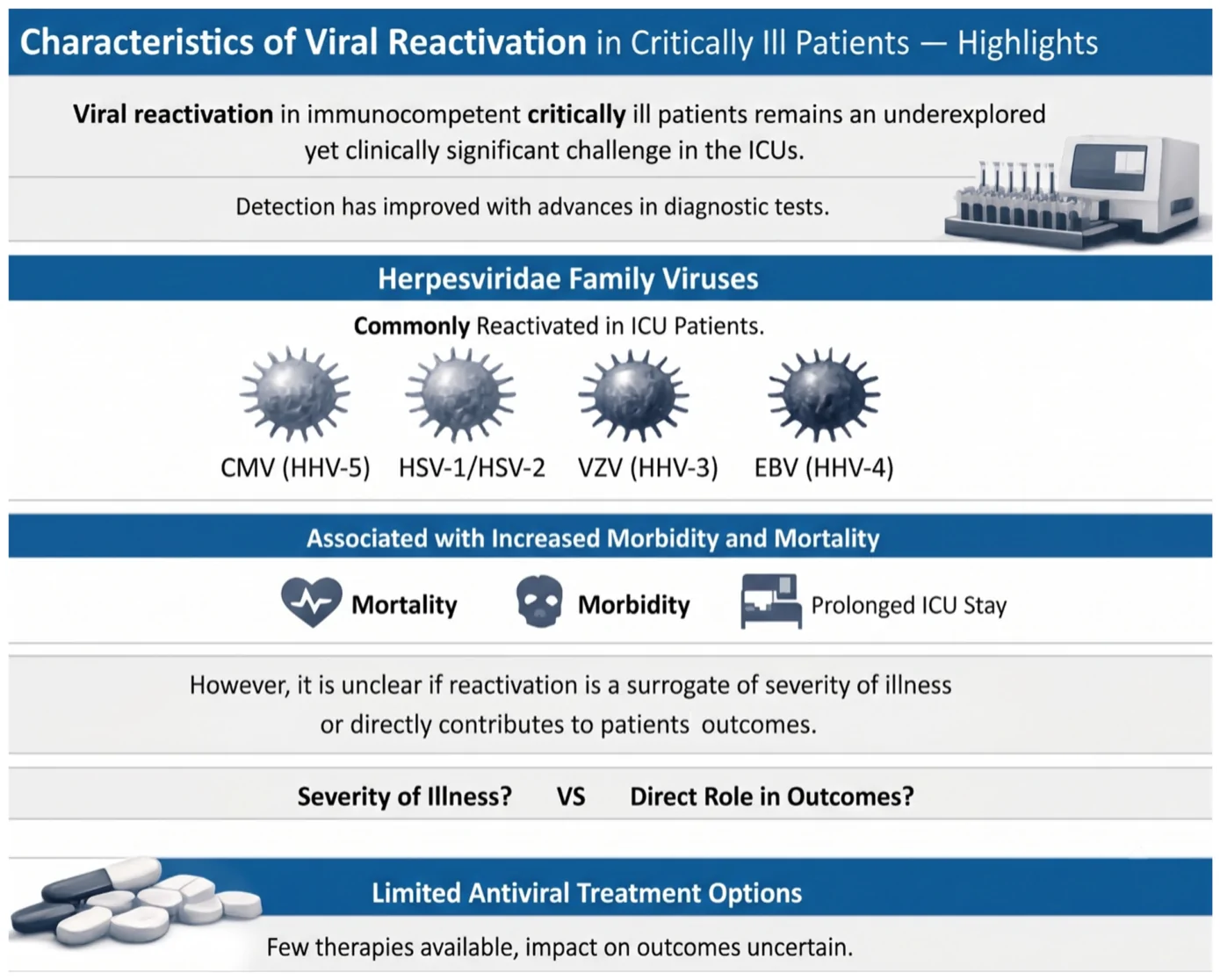
**HIGHLIGHTS**. Figure created with MS 365 Copilot based on the article’s conclusions.

In conclusion, viral reactivation in immunocompetent critically ill patients remains an underexplored yet clinically significant challenge in ICU settings. Although detection has improved due to advancements in diagnostic tests, it is still unclear how viral reactivation affects patient outcomes. Evidence suggests that reactivation correlates with worse clinical outcomes, including increased morbidity and mortality, although it is not clear that reactivation is the cause or a surrogate marker of underlying severity. Antiviral treatment options remain limited and the effectiveness of the outcomes is still uncertain. Addressing knowledge gaps through further research and refining management strategies is imperative to improve outcomes for this vulnerable population. Table 3 summarizes the main characteristics of viral reactivation in critically ill patients. Figure 1 depicts the article highlights.

## Figures and Tables

**Table 1:** Overt (classical) causes of immunosuppression according to EORTC/MSGERC criteria and ICU-specific adaptations.

Host Factors	Generic Population	ICU Population
**Neutropenia**	Neutropenia (<500 cells/mm^3^ for >10 days)	Qualitative or quantitative neutrophil dysfunction (including inherited neutrophil defects; ANC ≤ 500 cells/mm^3^)
**Transplantation**	HSCT; SOT	HSCT; SOT
**Corticosteroid exposure**	Prednisone ≥0.3 mg/kg for ≥3 weeks (past 60 days)	Prednisone ≥ 20 mg/day
**Comorbidities/co-infections**	Hematologic malignancy; acute GVHD grade III–IV refractory to steroids	Hematologic malignancy; decompensated cirrhosis; chronic airway disease; HIV; severe influenza or other severe viral pneumonia (including COVID-19).
**Immunodeficiency**	Primary immunodeficiencies (CGD, STAT3 deficiency, CARD9 deficiency, SCID, STAT1 GOF)	-
**Immunosuppressive therapies**	Calcineurin inhibitors, TNF-α blockers, lymphocyte-targeting monoclonal antibodies; nucleoside analogs during the past 90 days Treatment with recognized B-cell immunosuppressants, such as Bruton’s tyrosine kinase inhibitors, e.g., ibrutinib	Calcineurin/mTOR inhibitors; TNF-pathway inhibitors; alemtuzumab; ibrutinib; nucleoside analogs during the past 90 days
**ICU-related risk factors**		*Candida* colonization (≥2 sites) Parenteral nutrition
**Barrier impairment**		Impaired cutaneous barriers to bloodstream infection (CVC carriers, HD) Impaired gut wall integrity (recent abdominal surgery, chemotherapy, biliary disease, recurrent perforation, ascites, mucositis, severe pancreatitis)

The EORTC/MSGERC host factors associated with immunosuppression listed here refer to the risk of invasive candidiasis and aspergillosis infections acquisition, in line with the ICU population subanalysis performed by Bassetti et al. [4]; other pathogen-specific factors were not reported. ANC, absolute neutrophil count; CARD9 deficiency, caspase recruitment domain-containing protein 9 deficiency; CGD, chronic granulomatous disease; COVID-19, coronavirus disease 2019; CVC, central venous catheter; EORTC/MSGERC, European Organization for Research and Treatment of Cancer/Mycoses Study Group Education and Research Consortium; GOF, gain of function; HD, hemodialysis; HIV, human immunodeficiency virus infection; HSCT, allogeneic hematopoietic stem cell transplantation; mTOR, mammalian target of rapamycin; SCID, severe combined immunodeficiency; SOT, solid-organ transplant; STAT3 deficiency, signal transducer and activator of transcription 3 deficiency; TNF, tumor necrosis factor blockers.

**Table 2:** Non-overt (covert) causes of immunosuppression in the ICU.

Covert Causes of Immunosuppression in the ICU	
Acute acquired immunodeficiencies	Non-immune conditions
• Infections [5,6] ○ Sepsis [5,6] ○ Viral pneumonia (flu, SARS-CoV-2) [5,6] ○ Malaria [5,6] • Major trauma [5,6] • Major surgery [5,6] • Subarachnoid hemorrhage [5,6] • Cardiac arrest [5,6] • Duration of immunosuppression [6] • Persistent critical illness (PerCI) [6]	• Comorbidities [5,6] ○ Cirrhosis [5,6] ○ Diabetes [5,6] ○ Chronic pulmonary conditions (e.g., COPD) [5,6] ○ Obesity [7,8,9] • Duration of hospitalization (PICS: persistent inflammation, immunosuppression, and catabolism syndrome) [6,10]

**Table 3:** Viral reactivation in critically ill patients.

Virus	Reactivation (%)	Risk Factors	Clinical Consequences
**CMV (HHV-5)**	20–70% [29,31]	Sepsis, corticosteroids, immunosuppression	Pneumonitis, secondary infections, >mortality
**HSV 1,2**	20–60% (lesser in HSV-2) [49,51]	Sepsis, corticosteroids, immunosuppression	Pneumonia, rare mucocutaneous lesions, >ICU stay
**EBV (HHV-4)**	25–50% [57,58,59]	Sepsis, corticosteroids, immunosuppression, multi-organ failure	Pulmonary complications, increased inflammatory response
**VZV (HHV-3)**	1–5% [66,68]	Corticosteroids, immunosuppression, prior exposure to VZV, elderly	Disseminated infection, pulmonary complications

## Data Availability

No new data were created or analyzed in this study. Data sharing is not applicable to this article.
